# Chylous leakage after esophagectomy for esophageal cancer: a systematic review

**DOI:** 10.1186/s13019-024-02764-1

**Published:** 2024-04-17

**Authors:** Xing Zheng, Xi Yang, Sujuan Lei

**Affiliations:** 1 Department of Osteoarthrosis, Affiliated Hospital of Southwest Medical University, Luzhou, Sichuan China; 2 Department of Vascular Surgery, Affiliated Hospital of Southwest Medical University, Luzhou, Sichuan China; 3 Department of Hepatobiliary Surgery, Affiliated Hospital of Southwest Medical University, Luzhou, Sichuan China

**Keywords:** Esophagectomy, Chylous leakage, Neoplasms

## Abstract

**Background:**

Chylous leakage is a rare complication following esophagectomy; however, it can lead to mortality. We aimed to systematically evaluate the factors that may lead to increased chylous leakage after esophagectomy.

**Methods:**

Three databases (PubMed, Embase, and Cochrane Library) were systematically searched for all studies investigating the occurrence of chylous leakage after esophagectomy.

**Results:**

A total of 32 studies were identified, including 26 randomized controlled trials and 3 cohort and case–control studies, each. The overall incidence of chylous leakage was 4.7% (278/5,971 cases). Analysis of preoperative, intraoperative, and postoperative factors showed that most of the qualitative analysis results did not significantly increase the incidence of chylous leakage. In some quantitative analyses, the chylous leakage rate was significantly lower in the thoracic duct mass ligation group than in the conservative treatment group (relative risk [RR] = 0.33; 95% confidence interval [CI], 0.13–0.83; I^2^ = 0.0%; *P* = 0.327). Direct oral feeding significantly reduced chylous leakage compared with jejunostomy (RR = 0.06; 95% CI 0.01–0.33; I^2^ = 0.0%; *P* = 0.335). However, preoperative inspiratory muscle training (RR = 1.66; 95% CI, 0.21–12.33; I^2^ = 55.5%; *P* = 0.134), preoperative chemoradiotherapy (RR = 0.99; 95% CI, 0.55–1.80; I^2^ = 0.0%; *P* = 0.943), and robotic assistance (RR = 1.62; 95% CI, 0.92–2.86; I^2^ = 0.0%; *P* = 0.814) did not significantly reduce the incidence of chylous leakage.

**Conclusions:**

Ligation of the thoracic duct and direct oral feeding can reduce the incidence of chylous leakage after esophagectomy in patients with esophageal cancer. Other contributing factors remain unclear and require validation in further high-quality studies.

**Graphical Abstract:**

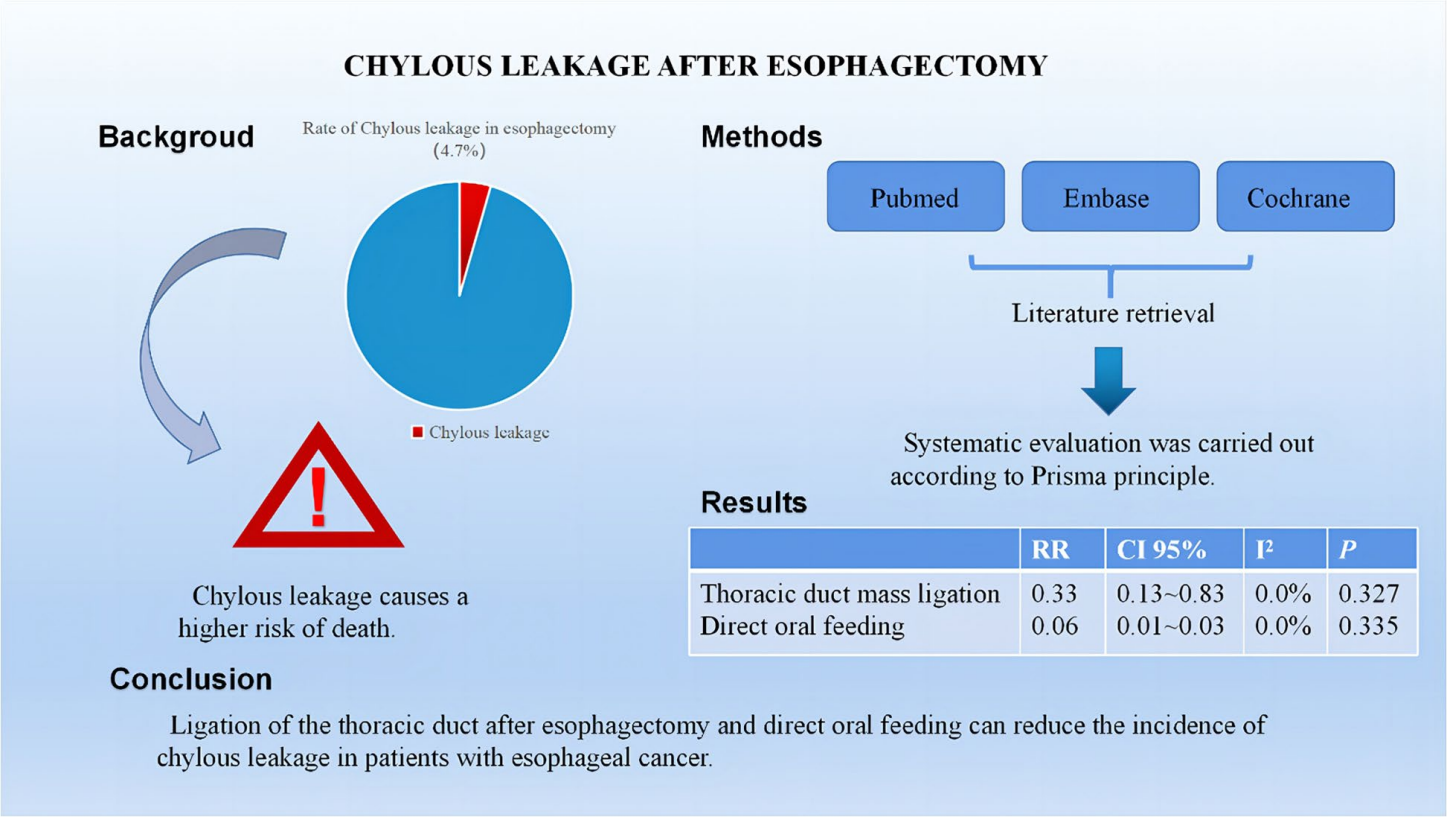

**Supplementary Information:**

The online version contains supplementary material available at 10.1186/s13019-024-02764-1.

## Background

Postesophagectomy chylous leakage is a rare but fatal complication with an incidence rate of 0.4–9% following transthoracic esophagectomy [1]. Daily chylous leakage after surgery can result in dehydration, electrolyte abnormalities, malnutrition, and lymphocytopenia, which increase the risk of mortality [2].

Chylous leakage is defined as the postoperative drainage of a milky fluid rich in triglycerides; however, the threshold volume to define chylous leakage is unclear: some authors accepted 110 mg/dL triglycerides as a threshold, while others preferred 200 mg/dL [3]. The optimal management strategy for chylous leakage remains unclear. In 70% of patients, chylous leakage can be cured through conservative methods such as negative pressure drainage, pressure dressing, somatostatin, and a low-fat diet [4]. For patients with persistently high output or those who are unresponsive to conservative treatment, further treatment options include surgical exploration of the wound, thoracoscopic thoracic catheter ligation, and percutaneous embolization [5].

Thoracic catheter ligation has been indicated as an effective measure to reduce the incidence of postoperative chylothorax, and can be administered prophylactically [6]. Two other studies reported a lower incidence of chylorrhagia after esophagectomy in patients with high body mass index [7, 8]. According to the 2022 international consensus statement [9], there is still a lack of standard management and treatment options for these cases [10]. This study used a systematic review to search the literature databases for original studies investigating the occurrence of chylous leakage after esophagectomy for esophageal cancer. Based on an analysis of these studies, we summarized the various management methods applied before, during, and after surgery, and comprehensively explored the prevention and treatment measures for chylorrhea other a single surgical method or postoperative treatment. To provide a comprehensive and rapid reference for the prevention and treatment of chylous leakage, this study systematically summarized and analyzed existing studies of the occurrence of chylous leakage after esophagectomy.

## Methods

### Study retrieval

The PubMed, Embase, and Cochrane Library databases were searched from inception to December 30, 2022, for all relevant studies without language restrictions. Specific search terms included “Esophagectomy/Esophagectomies” and “Intraoperative Complications/Postoperative Complications.” The three databases were systematically searched using combinations of the above search terms and Medical Subject Headings synonyms.

### Inclusion and exclusion criteria

#### Inclusion criteria

Participants: Patients with esophageal cancer undergoing esophagectomy. Complications: All studies reporting rates of chylous leakage after esophagectomy.

### Exclusion criteria

Duplicate studies (the most comprehensive study was retained); case reports, summaries, and conference abstracts; and studies of postesophagectomy complications without chylous leakage.

### Literature screening

The literature was independently searched by two researchers, and the identified references were imported into Endnote ver. 20 software to build the study database. Duplicate references were eliminated through review, and the retrieved articles were subjected to title and abstract screening. All remaining articles were subjected to full-text review. In cases of disagreement, a third researcher arbitrated until consensus was reached.

### Quality evaluation

The quality of the enrolled studies was independently evaluated by two researchers using authenticity evaluation tools, with the results discussed after completion. In cases of disagreement, a third party was consulted again, and the issue was discussed until a consensus was reached.

#### Randomized controlled trials

The quality of the included randomized controlled trials (RCTs) was evaluated using the 2016 JBI Evidence-Based Health Care Center of Australia’s RCTs Authenticity Evaluation tool [11]. The quality assessment tool features four options for each evaluated item: yes, no, unclear, and not applicable. The 13 items included whether (1) random grouping was adopted for the research participants; (2) the distribution was hidden; (3) the baseline variables were comparable between groups; (4) the participants were blinded; (5) the intervention was blinded; (6) the evaluator was blinded; (7) the groups received the same measures other than the tested intervention; (8) the follow-up was complete and, if not, whether measures were taken to manage loss to follow-up; (9) all randomly assigned research objects were included in the results analysis; (10) the outcome indicators of each group were identically evaluated; (11) the outcome index evaluation method was credible; (12) the data analysis method was appropriate; and (13) the study design was reasonable. Any differences in the conduct of research and data analysis among the RCTs was further assessed.

#### Case–control studies

The authenticity of the case–control studies was also evaluated using the JBI tool [11]. The 10 measurement items for these studies investigated whether (1) other factors were comparable between the case and control groups, except for disease status; (2) matching was appropriate between the case and control groups; (3) the same criteria were used to recruit the case and control groups; (4) standard, effective, and credible methods were used to assess exposure factors; (5) the exposure factors of the case and control groups were identically assessed; (6) confounding factors were considered; (7) confounding factors were controlled for; (8) standard, effective, and credible methods were used to evaluate outcome indicators; (9) exposure time was sufficient; and (10) the data analysis method was appropriate.

#### Cohort study

All cohort studies were also evaluated using the JBI tool [11]. The 11 items included in this evaluation investigated whether (1) the research participants of each group had similar characteristics and were derived from the same research population; (2) the exposure factors were identically assessed and the participants were assigned to the exposed versus nonexposed group; (3) the evaluation method of exposure factors was effective and credible; (4) confounding factors were considered; (5) confounding factors were controlled for; (6) the absence of observed outcomes in the participants at exposure or the study start were described; (7) the outcome index evaluation method was effective and credible; (8) the follow-up time was reported and the follow-up time was sufficient to observe the occurrence of outcome indicators; (9) the follow-up was complete and, if not, the reasons for loss to follow-up were described and analyzed; (10) measures were taken to manage loss to follow-up; and (11) the data analysis method was appropriate.

### Data extraction

A data extraction table was designed in advance, and relevant data were extracted and crosschecked by two researchers who performed an independent review of the studies. The extracted data included first author, year of publication, country of origin, intervention, subject ages, number of study participants, number of patients with chylous leakage in the intervention versus control group, main results, and conclusions. In cases of disagreement, the third party intervened until a consensus was reached. Missing important information was obtained from the original author whenever possible.

### Data analysis

The studies enrolled in this analysis differed significantly in terms of research design, content, and methods and other aspects. As such, a qualitative analysis was mainly adopted to systematically summarize and describe the occurrence of chylous leakage in the included studies. Part of the extracted data was analyzed using Stata 15. Based on the interstudy heterogeneity, the Higgins index (I^2^) was used to quantitatively evaluate the results using a combination of fixed- or random-effects models. Studies were divided into low-, medium-, and high-heterogeneity groups based on the I^2^ values using cutoffs of 50% and 75%, respectively. When heterogeneity was low, a fixed-effects model was used to merge the data; when the heterogeneity was high, a subgroup or sensitivity analysis was needed, and the data were combined using a random-effects model. All tests were two-tailed, and *P* < 0.05 was considered statistically significant.

## Results

### Literature search and screening results

**Fig. 1:**
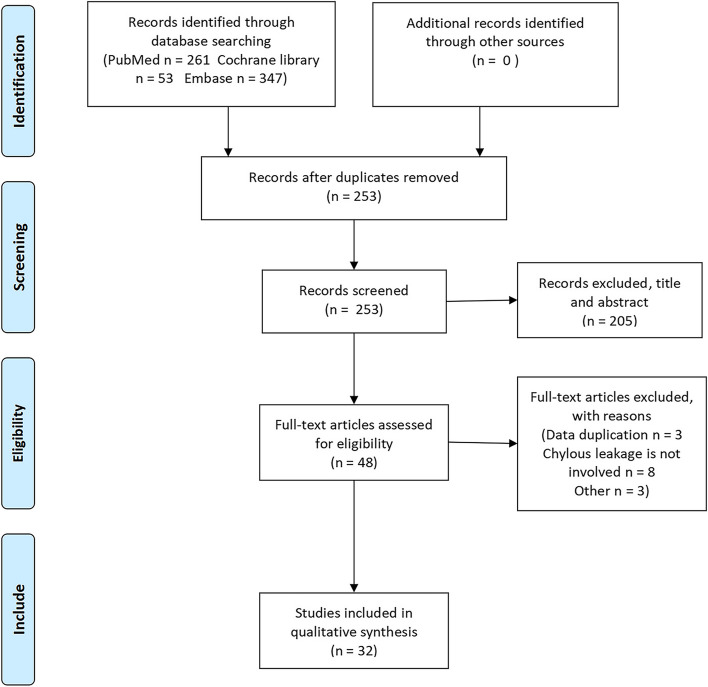
Flow chart of the study retrieval process

Searches of the PubMed (*n* = 261), Embase (*n* = 347), and Cochrane Library (*n* = 53) yielded 661 studies. Of these, 253 were retained after the initial screening; 48 articles remained after title and abstract screening; and 32 studies remained after full-text screening, including 26 RCTs, 3 cohort studies, and 3 case–control studies. The selection process is illustrated in Fig. 1.

### Inclusion characteristics

The 32 included studies were published between 2003 and 2022 in India (*n* = 1), China (*n* = 12), the Netherlands (*n* = 9), France (*n* = 1), Germany (*n* = 2), the United Kingdom (*n* = 1), Japan (*n* = 5), and Iran (*n* = 1). The specific occurrences of chylous leakage are listed in Table 1.

**Table 1:** Occurrence of chylous leakage among the included studies

Author	Year	Country	Experimental	Number of chylous leakage cases/total cases	Control	Number of chylous leakage cases/total cases	Age
Hayes N	1995	Britain	Lewis-Tanner two-stage	1/14	Synchronous two-team	0/13	63(40–74) 68(51–77)
Bruns H	1996	Germany	Transthoracic en bloc resection	0/12	Transmediastinal dissection	1/10	58(46–69)
Lanschot	1999	Holland	Prevertebral gastric tube reconstruction	3/30	Retrosternal gastric tube reconstruction	2/30	60(37–76) 63(43–79)
Han-Geurts IJM	2007	Holland	Feeding jejunostomy	0/79	Nasoduodenal tube placement	2/71	61(28–89) 61(39–85)
Hirao M	2011	Japan	Preoperative chemotherapy	1/162	Postoperative chemotherapy	2/154	61(39–75) 61(38–75)
Lai FC	2011	China	Mass Ligation of Thoracic Duct	1/325	Conserved group	7/328	68.6 ± 9.4 67.4 ± 9.0
Nederlof N	2011	Holland	End-to-end esophagogastrostomy	4/64	End-to-side esophagogastrostomy	1/64	60 [35–80] 63 [39–82]
Zhang C	2011	China	Narrow gastric tube reconstruction	0/52	Total gastric reconstruction	1/52	91 of them(51–70)
Li B	2015	China	Sweet Esophagectomy	11/150	Ivor-Lewis Esophagectomy	5/150	60 (39–74) 60 (38–74)
Mashhadi MR	2015	Iran	Preoperative radiochemotherapy	2/50	Surgical treatment	1/50	56.0 ± 5.62 57.7 ± 3.80
Zhang Z	2017	China	Chemoradiotherapy + surgery	11/141	Chemotherapy + surgery	10/126	61 (44–69) 62 (46–72)
Guinan EM	2018	Holland	Preoperative inspiratory muscle training	3/28	Standard treatment	0/32	63.07 (8.8) 65.06(7.78)
Yang H	2018	China	Preoperative chemoradiotherapy + surgery	5/185	Surgical treatment	7/227	56 (31–70) 58 (35–70)
Ohkura Y	2018	Japan	Oligomer formula, enteral nutrition	3/33	Polymer formula, enteral nutrition	1/34	(≥ 65/ < 65)37/30
Pieter C	2018	Holland	Robot-assisted Minimally Invasive Thoracolaparoscopic Esophagectomy	17/54	Open Transthoracic Esophagectomy	12/55	64 (± 8.9) 65 (± 8.2)
Kanekiyo S	2018	Japan	Immunomodulatory enteral nutrition	1/20	Standard enteral nutrition	1/20	65 (60–70) 62 (60–72)
Valkenet K	2018	Holland	Preoperative inspiratory muscle training	11/120	Standard treatment	13/118	62.7(8.9) 63.7(7.5)
Li B	2019	China	Three-field lymphadenectomy in transthoracic esophagectomy	7/200	Two-field lymphadenectomy in transthoracic esophagectomy	7/200	62 (57–66) 61 (57–66)
Berkelmans GHK	2019	Holland	Direct Oral Feeding Following Minimally Invasive Esophagectomy	1/65	Feeding jejunostomy	7/67	65 [59–70] 65 [61–70]
Mariette C	2019	France	Open surgery	7/103	Mixed minimally invasive surgery	5/102	61(23–78) 59(23–75)
Liu B	2019	China	Modified gastric tube	1/35	Conventional gastric tube	1/35	64.06 ± 8.69 65.00 ± 10.12
Zheng T	2019	China	Nasojejunostomy feeding	0/58	Nasogastric feeding	0/62	65 (41–81) 64 (39–82)
Sasaki K	2020	Japan	Postoperative large-curvature anastomosis	1/35	Postoperative small-curvature anastomosis	0/33	65 (46–80) 65 (51–75)
Fransen LFC	2020	Holland	Direct Oral Feeding Following Minimally Invasive Esophagectomy	0/85	feeding jejunostomy	21/111	65 (58–70) 67 (61–74)
Sugimura K	2020	Japan	Chemoradiotherapy	2/40	Chemotherapy	1/41	65 (43–79) 67.5(50–76)
Zhong JD	2021	China	Postoperative active respiratory circulation technique	2/146	Regular chest physical therapy	2/145	61.2(8.61) 61.1(8.25)
Shi KF	2021	China	Video-assisted mediastinoscopic Esophagectomy	1/100	Laparoscopic transhiatal Esophagectomy	4/100	66.3 ± 6.1 66.3 ± 6.7
Yang Y	2021	China	Robot-assisted + minimally invasive surgery	5/181	Conventional minimally invasive surgery	2/177	65 (43–75) 63(42–75)
Workum FV	2021	Holland	Minimally invasive with intrathoracic anastomosis	9/122	Minimally invasive cervical anastomosis	11/123	67 (5.1) 68 (9.2)
Kulkarni A	2022	India	Robot-assisted McKeown esophagectomy	2/25	Video-assisted McKeown esophagectomy	2/49	59.2 ± 8.3 56.1 ± 11.1
Fabbi M	2022	Germany	Cycle stapler, end-to-side	26/220	Linear stapler, side-to-side	5/36	66(36–89) 65(29–83)
Wang H	2022	China	Chemoradiotherapy + surgery	3/114	Chemotherapy + surgery	3/108	18–75

### Study quality evaluation

Thirty-two articles were included, including 26 RCTs and 3 case–control and cohort studies, each. Specific quality evaluation results are presented in Supplementary Tables 1, 2 and 3.

### Chylous leakage after esophagectomy

#### Preoperative

**Fig. 2:**
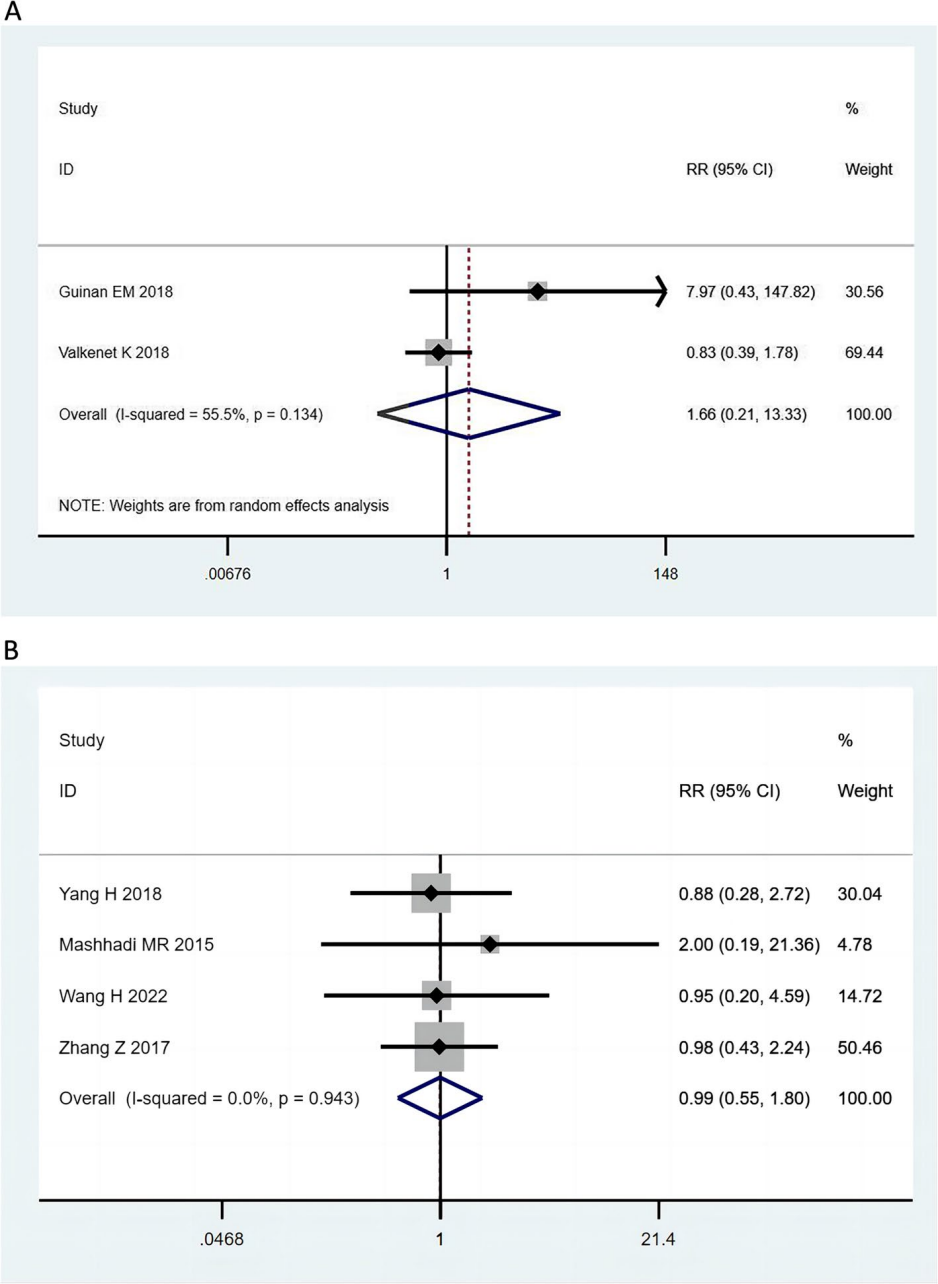
Preoperative. **A** Preoperative inspiratory muscle training did not reduce the risk of chylous. **B** Chemoradiotherapy did not reduce the risk of chylous before surgery

Two studies reported on inspiratory muscle training before esophagectomy, with analysis indicating no significant effect on the occurrence of chylous leakage (RR = 1.66; 95% CI, 0.21–12.33; I^2^ = 55.5%; *P* = 0.134) (Fig. 2A), with moderate heterogeneity. A fixed-effect model was used to combine the results. Four studies reported that preoperative chemoradiotherapy (RR = 0.99; 95% CI, 0.55–1.80; I^2^ = 0.0%; *P* = 0.943) (Fig. 2B) did not differ significantly in the occurrence of chylous leakage. A fixed-effects model was used to combine the results.

### Choice of surgical method

**Fig. 3:**
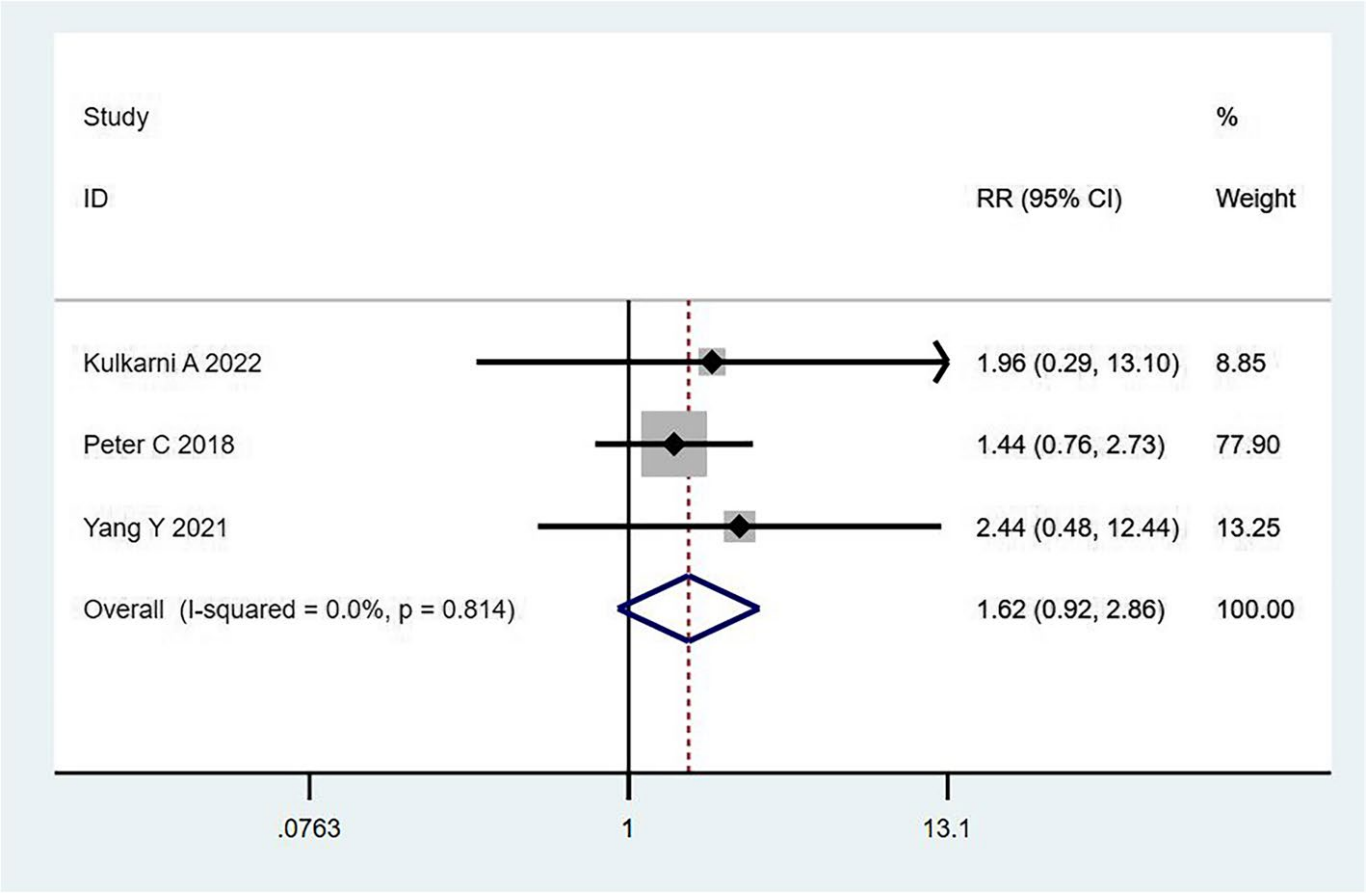
Robot-assisted surgery did not reduce the risk of chylous

Three studies reported that robot-assisted minimally invasive surgery did not significantly reduce the incidence of chylous leakage (RR = 1.62; 95% CI, 0.92–2.86; I^2^ = 0.0%; *P* = 0.814) (Fig. 3), and no significant heterogeneity was observed. A fixed-effects model was used to combine the results. No significant difference in the incidence of chylous leakage was noted between patients treated with mediastinal esophageal dissection and thoracic holistic esophagectomy, Sweet and Ivor–Lewis surgery, open and mixed minimally invasive surgery, or video-assisted mediastinal laparoscopic and thoracoscopic surgery.

### Intraoperative factors

**Fig. 4:**
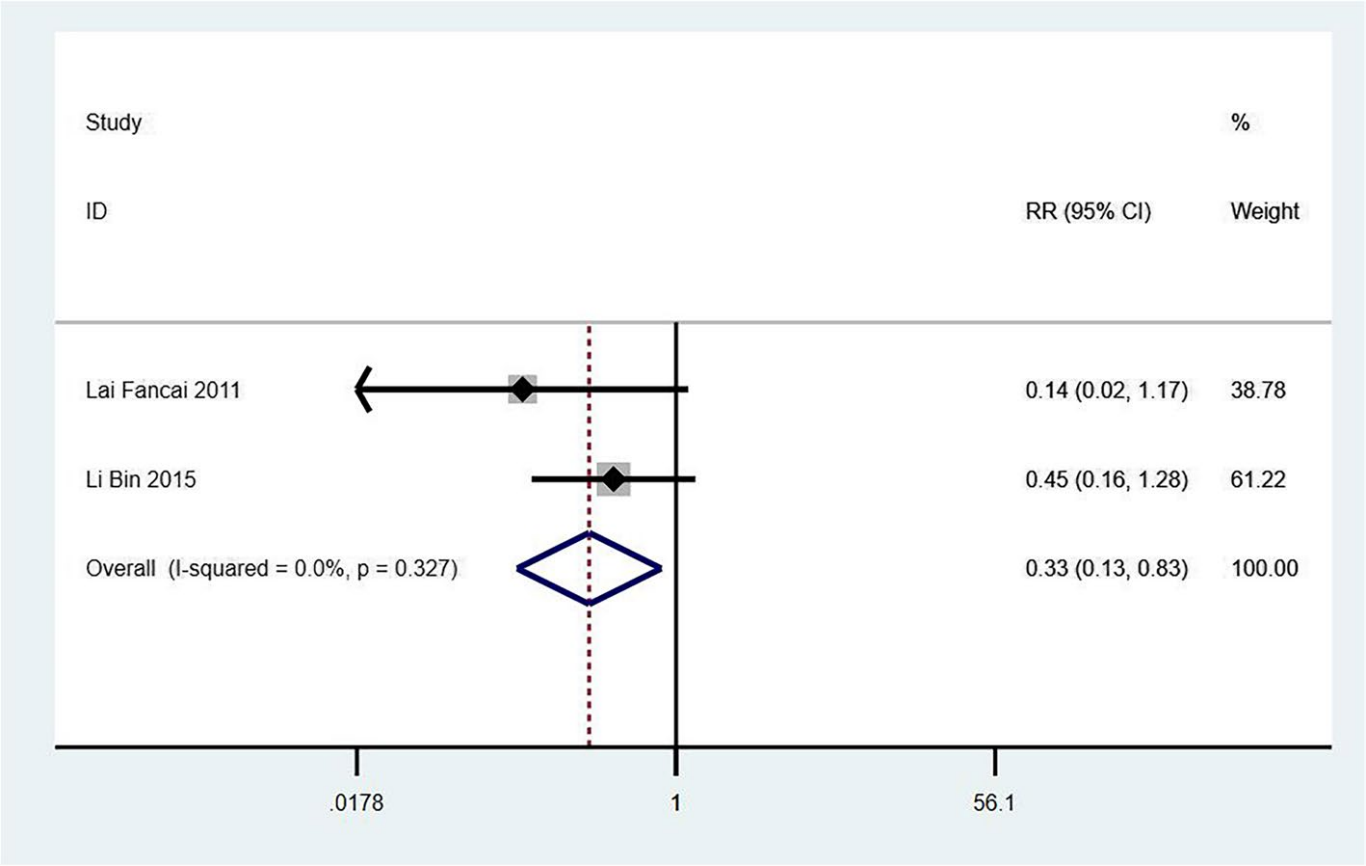
Thoracic catheter ligation reduces the risk of chylous

The influences of postoperative prevertebral versus retrosternal gastric canal reconstruction, jejunostomy versus nasoduodenal anastomosis, postoperative end-to-end versus end-to-side anastomosis, narrow versus total gastric canal reconstruction, three-stage versus two-end lymphatic dissection, modified versus conventional gastric canal, minimally invasive with intrathoracic versus cervical anastomosis, circular versus linear stapler end-to-side anastomosis, and postoperative large-curvature versus small-curvature anastomosis were compared. No significant difference was observed in the occurrence of chylous leakage between anastomosis and small curvature anastomosis. Chylous leakage was significantly reduced in the thoracic duct mass ligation group versus the conservative treatment group (RR = 0.33; 95% CI, 0.13–0.83; I^2^ = 0.0%; *P* = 0.327) (Fig. 4), and no obvious heterogeneity was observed. The fixed-effect model was used to combine the results.

### Management in the convalescent stage

**Fig. 5:**
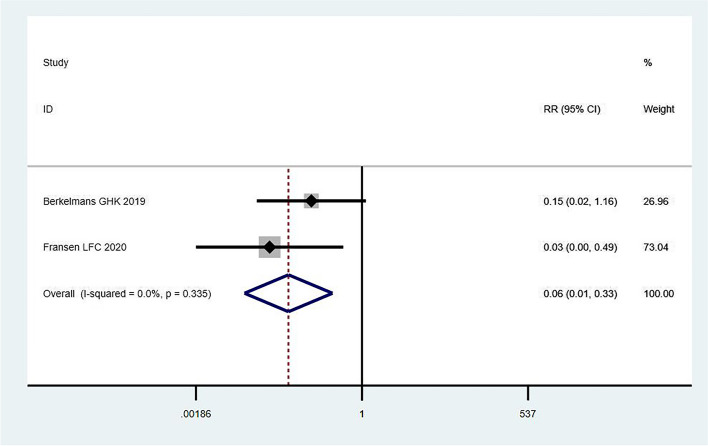
Direct oral feeding reduces the risk of chylous

Postoperative versus preoperative chemotherapy, oligoformula versus polymer formula enteral nutrition, immunomodulatory versus standard enteral nutrition, nasojejunostomy versus naso-stomach feeding, and postoperative active respiratory circulation therapy versus conventional chest physical therapy were further investigated, revealing no significant differences in the occurrence of chylous leakage. Chylous leakage was significantly reduced by direct oral feeding compared with jejunostomy (RR = 0.06; 95% CI, 0.01–0.33; I^2^ = 0.0%; *P* = 0.335) (Fig. 5) without significant heterogeneity. A fixed-effects model was used to combine the results.

## Discussion

This study included predominantly RCTs (*n* = 26), with several case–control studies (*n* = 3) and cohort studies (*n* = 3). The aim of this study was to summarize the results existing studies of investigating chylous leakage after esophagectomy to explore the causes of chylous leakage. We included a large number of studies on chylous leakage after esophagectomy involving various management methods before, during, and after esophagectomy, but the incidence of chylous leakage was relatively small (the number of cases/total cases was 278/5,971, approximately 4.7%). A quantitative analysis of our limited data showed that thoracic catheter ligation and direct oral feeding after esophagectomy significantly reduced the incidence of chylous leakage. Although thoracic catheter ligation can significantly reduce the occurrence of chylous leakage, it is a traumatic procedure that can increase stress and is associated with risk such as catheter rupture, adversely affecting the patient’s immunity, nutritional status, and survival [12]. However, after esophagectomy, chylothorax can lead to hypovolemia, metabolic and nutritional depletion, and infection, and has a mortality rate exceeding 50% if untreated, with surgical ligation of thoracic catheterization generally considered the most appropriate treatment [13]. According to the current study by Berkelmans [14], fear of complications is the primary reason for delayed oral ingestion in patients undergoing esophagectomy, but the timing of direct oral ingestion after surgery does not lead to a higher incidence or more serious complications. Our pooled analysis showed that the incidence of chylous leakage was significantly reduced by direct oral ingestion postoperatively.

We further attempted to analyze the utility of inspiratory muscle training before esophagectomy and found no significant difference in the incidence of chylous leakage between patients with and without preoperative inspiratory muscle training. Preoperative inspiratory muscle training, which increases inspiratory muscle strength and endurance, did not reduce the incidence of postoperative pneumonia or significantly affect the occurrence of chylous leakage in patients following esophagectomy for esophageal cancer [15]. In addition, compared with patients who had received preoperative radiotherapy and chemotherapy followed by surgery, the survival rate of patients with locally advanced esophageal cancer was improved, the adverse events were acceptable and manageable, with no significant change in the incidence of chylous leakage [16].

Regarding the selection of surgical methods, the complication rate of synchronous double-approach esophagectomy was higher than that of conventional surgery, and Lewis–Tanner two-stage esophagectomy was recommended for patients with esophageal cancer [17]. Ivor–Lewis surgery may be associated with a lower postoperative complication rate and less lymphatic leakage [18], while Ivor–Lewis and Sweet esophagectomy are both safe surgical methods. Compared to direct thoracotomy and transthoracic surgery, robot-assisted minimally invasive combined thoracotomy is associated with a lower incidence of overall surgery-related and cardiopulmonary complications, less postoperative pain, better short-term quality of life, and better postoperative short-term functional recovery. The oncology results were comparable and in line with current standards [19]. Compared with open esophagectomy, mixed minimally invasive esophagectomy reduced the incidence of major intra- and postoperative complications, especially pulmonary complications, and did not affect 3-year overall and disease-free survival [20]. Generally, no significant difference is observed in the occurrence of chylous leakage among different surgical methods; therefore, we propose that the choice of surgical method does not affect the occurrence of postoperative chylous leakage in patients with esophageal cancer.

This study included prevertebral and retrosternal gastric canal reconstruction, jejunostomy and nasoduodenal anastomosis, postoperative end-to-end or end-to-side anastomosis, narrow gastric canal reconstruction and total gastric reconstruction, three-field and two-field lymphatic dissection, modified gastric canal and conventional gastric canal, minimally invasive and minimally invasive cervical anastomosis, circular and linear stapler, and postoperative large-curvature anastomosis consistent with the small curvature. No significant difference in the occurrence of chylous leakage was observed [21–29].

Perioperative immunonutrition can help to improve early nutritional status and reduce postoperative infection complications among patients undergoing esophageal cancer resection [30]. Compared to nasogastric feeding, nasojejunostomy offers greater safety, efficacy, and practicality for minimally invasive McKeown esophagostomy in patients with a high incidence of anastomotic leakage; however, its high risk of postoperative intestinal obstruction requires significant attention [31]. The postoperative active respiratory circulation technique can significantly reduce the incidence of complications associated with esophagectomy [32]. Our study shows that direct oral feeding can achieve a lower incidence of chylorrhagia than jejunostomy. This may be related to wound healing and impaired immune function, while direct oral feeding could be more beneficial for the gastrointestinal flora and mucosal immune disorders, thus reducing acute phase reactions. In addition, intraoperative damage to the lymphatic vessels may result in an abnormal increase in chylous fluid, which increases chylous leakage in the chest [33].

This study lacked sufficient data to perform a pooled analysis and the sample size was small; however, the quality of the included studies was relatively high and most studies were RCTs; therefore, the results have a certain credibility. Thoracic catheter ligation and postoperative direct oral feeding can reduce the risk of chylous leakage. However, in terms of the selection of preoperative chemoradiotherapy, esophagectomy surgical method, and postoperative recovery measures, whether preoperative chemoradiotherapy affects the occurrence of chylous leakage remains unclear. In addition, although a large number of prior studies have reported the occurrence of chylorrhea after esophagectomy, the majority are relatively simple, and there is currently a lack of special studies on chylorrhea. As such, more relevant studies should be performed in the future to provide more accurate and comprehensive scientific evidence to identify better prevention and treatment methods for chylorrhea after esophagectomy.

### Supplementary Information

**Supplementary Material 1.**

## Data Availability

Supplementary Data mentioned in the text are available.
