# Supplementary material for: Chylous leakage after esophagectomy for esophageal cancer: a systematic review
Source: J Cardiothorac Surg. 2024 Apr 17;19:240. doi: 10.1186/s13019-024-02764-1 (PMC11022397; doi:10.1186/s13019-024-02764-1)
Supplement: Supplementary file 1 — Supplementary Material 1. [file 13019_2024_2764_MOESM1_ESM.docx]

Supplementary Table 1 Quality evaluation of RCTs.

| **RCTs** | **①** | **②** | **③** | **④** | **⑤** | **⑥** | **⑦** | **⑧** | **⑨** | **⑩** | **⑪** | **⑫** | **⑬** |
| --- | --- | --- | --- | --- | --- | --- | --- | --- | --- | --- | --- | --- | --- |
| Lanschot 1999[1] | Yes | Unclear | Yes | Unclear | Unclear | Unclear | Yes | Yes | Yes | Yes | Yes | Yes | No |
| Han-Geurts IJM 2007[2] | Yes | Yes | Yes | Yes | Yes | Unclear | Yes | Yes | Yes | Yes | Yes | Yes | Yes |
| Hirao M 2011[3] | Yes | No | Yes | No | No | No | Yes | Yes | Yes | Yes | Yes | Yes | Yes |
| Lai FC 2011[4] | Yes | Unclear | Yes | Unclear | Unclear | Unclear | Yes | Yes | Yes | Yes | Yes | Yes | Yes |
| Nederlof N 2011[5] | Yes | Yes | Yes | No | No | No | Yes | Yes | Yes | Yes | Yes | Yes | Yes |
| Zhang C 2011[6] | Yes | Yes | Yes | Yes | Yes | Yes | Yes | Yes | Yes | Yes | Yes | Yes | Yes |
| Li B 2015[7] | Yes | Yes | Yes | Yes | No | No | Yes | Yes | Yes | Yes | Yes | Yes | Yes |
| Mashhadi MR 2015[8] | Yes | Unclear | Yes | Unclear | Unclear | Unclear | Yes | Yes | Yes | Yes | Yes | Yes | Yes |
| Zhang Z 2017[9] | Yes | Unclear | Yes | Unclear | Unclear | Unclear | Yes | Yes | Yes | Yes | Yes | Yes | Yes |
| Guinan EM 2018[10] | Yes | No | Yes | Yes | No | No | Yes | Yes | Yes | Yes | Yes | Yes | Yes |
| Yang H 2018[11] | Yes | Yes | Yes | Yes | Yes | No | Yes | No | Yes | Yes | Yes | Yes | Yes |
| Ohkura Y 2018[12] | Yes | No | Yes | Yes | No | No | Yes | Yes | Yes | Yes | Yes | Yes | Yes |
| Pieter C 2018[13] | Yes | Yes | Yes | Yes | No | No | Yes | Yes | Yes | Yes | Yes | Yes | Yes |
| Kanekiyo S 2018[14] | Yes | Yes | Yes | Yes | No | No | Yes | Yes | Yes | Yes | Yes | Yes | Yes |
| Valkenet K 2018[15] | Yes | Yes | Yes | Yes | No | Yes | Yes | Yes | Yes | Yes | Yes | Yes | Yes |
| Li B 2019[16] | Yes | Yes | Yes | No | No | No | Yes | Yes | Yes | Yes | Yes | Yes | Yes |
| Berkelmans GHK 2019[17] | Yes | Yes | Yes | Unclear | Unclear | Unclear | Yes | No | Yes | Yes | Yes | Yes | Yes |
| Mariette C 2019[18] | Yes | Yes | Yes | Yes | Yes | Yes | Yes | Yes | Yes | Yes | Yes | Yes | Yes |
| Zheng T 2019[19] | Yes | No | Yes | Yes | No | No | Yes | Yes | Yes | Yes | Yes | Yes | Yes |
| Sasaki K 2020[20] | Yes | Yes | Yes | Unclear | Unclear | Unclear | Yes | Yes | Yes | Yes | Yes | Yes | Yes |
| Sugimura K 2020[21] | Yes | Yes | Yes | Yes | No | No | Yes | No | Yes | Yes | Yes | Yes | Yes |
| Zhong JD  2021[22] | Yes | Yes | Yes | Yes | Yes | Yes | Yes | Yes | Yes | Yes | Yes | Yes | Yes |
| Shi KF 2021[23] | Yes | Yes | Yes | No | No | No | Yes | Yes | Yes | Yes | Yes | Yes | Yes |
| Yang Y 2021[24] | Yes | Yes | Yes | Unclear | Unclear | Unclear | Yes | Yes | Yes | Yes | Yes | Yes | Yes |
| Workum FV 2021[25] | Yes | Yes | Yes | Yes | Yes | Yes | Yes | Yes | Yes | Yes | Yes | Yes | Yes |
| Wang H 2022[26] | Yes | Yes | Yes | Yes | Yes | Yes | Yes | No | Yes | Yes | Yes | Yes | Yes |

Supplementary Table 2 Quality evaluation of case-control studies.

| **Case-control studies** | **①** | **②** | **③** | **④** | **⑤** | **⑥** | **⑦** | **⑧** | **⑨** | **⑩** |
| --- | --- | --- | --- | --- | --- | --- | --- | --- | --- | --- |
| Liu B 2019[27] | Yes | Yes | Yes | Yes | Yes | No | No | Yes | Yes | Yes |
| Kulkarni A 2022[28] | Yes | Yes | Yes | Yes | Yes | No | No | Yes | Yes | Yes |
| Fabbi M 2022[29] | Yes | Yes | Yes | Yes | Yes | Yes | Yes | Yes | Yes | Yes |

Supplementary Table 3 Quality evaluation of cohort studies.

| **Cohort studies** | **①** | **②** | **③** | **④** | **⑤** | **⑥** | **⑦** | **⑧** | **⑨** | **⑩** | **⑪** |
| --- | --- | --- | --- | --- | --- | --- | --- | --- | --- | --- | --- |
| Hayes N 1995[30] | Yes | Yes | Yes | No | No | No | Yes | Yes | Yes | Unclear | Yes |
| Bruns H 1996[31] | Yes | Yes | Yes | No | No | No | Yes | Yes | Yes | Unclear | Yes |
| Fransen LFC 2020[32] | Yes | Yes | Yes | Yes | Yes | Yes | Yes | Unclear | Yes | No | Yes |

1. van Lanschot JJ, van Blankenstein M, Oei HY, Tilanus HW: Randomized comparison of prevertebral and retrosternal gastric tube reconstruction after resection of oesophageal carcinoma. Br J Surg 1999, 86(1):102-108.

2. Han-Geurts IJ, Hop WC, Verhoef C, Tran KT, Tilanus HW: Randomized clinical trial comparing feeding jejunostomy with nasoduodenal tube placement in patients undergoing oesophagectomy. Br J Surg 2007, 94(1):31-35.

3. Hirao M, Ando N, Tsujinaka T, Udagawa H, Yano M, Yamana H, Nagai K, Mizusawa J, Nakamura K, Japan Esophageal Oncology Group/Japan Clinical Oncology G: Influence of preoperative chemotherapy for advanced thoracic oesophageal squamous cell carcinoma on perioperative complications. Br J Surg 2011, 98(12):1735-1741.

4. Lai FC, Chen L, Tu YR, Lin M, Li X: Prevention of chylothorax complicating extensive esophageal resection by mass ligation of thoracic duct: a random control study. Ann Thorac Surg 2011, 91(6):1770-1774.

5. Nederlof N, Tilanus HW, Tran TC, Hop WC, Wijnhoven BP, de Jonge J: End-to-end versus end-to-side esophagogastrostomy after esophageal cancer resection: a prospective randomized study. Ann Surg 2011, 254(2):226-233.

6. Zhang C, Wu QC, Hou PY, Zhang M, Li Q, Jiang YJ, Chen D: Impact of the method of reconstruction after oncologic oesophagectomy on quality of life--a prospective, randomised study. Eur J Cardiothorac Surg 2011, 39(1):109-114.

7. Li B, Xiang J, Zhang Y, Li H, Zhang J, Sun Y, Hu H, Miao L, Ma L, Luo X et al: Comparison of Ivor-Lewis vs Sweet esophagectomy for esophageal squamous cell carcinoma: a randomized clinical trial. JAMA Surg 2015, 150(4):292-298.

8. Rajabi Mashhadi M, Bagheri R, Abdollahi A, Ghamari MJ, Shahidsales S, Salehi M, Shahkaram R, Majidi MR, Sheibani S: The Effect of Neoadjuvant Therapy on Early Complications of Esophageal Cancer Surgery. Iran J Otorhinolaryngol 2015, 27(81):279-284.

9. Zhang Z, Zhang H: Impact of neoadjuvant chemotherapy and chemoradiotherapy on postoperative cardiopulmonary complications in patients with esophageal cancer. Dis Esophagus 2017, 30(4):1-7.

10. Guinan EM, Forde C, O'Neill L, Gannon J, Doyle SL, Valkenet K, Trappenburg JCA, van Hillegersberg R, Ravi N, Hussey JM et al: Effect of preoperative inspiratory muscle training on physical functioning following esophagectomy. Dis Esophagus 2019, 32(2).

11. Yang H, Liu H, Chen Y, Zhu C, Fang W, Yu Z, Mao W, Xiang J, Han Y, Chen Z et al: Neoadjuvant Chemoradiotherapy Followed by Surgery Versus Surgery Alone for Locally Advanced Squamous Cell Carcinoma of the Esophagus (NEOCRTEC5010): A Phase III Multicenter, Randomized, Open-Label Clinical Trial. J Clin Oncol 2018, 36(27):2796-2803.

12. Ohkura Y, Ueno M, Shindoh J, Iizuka T, Udagawa H: Randomized controlled trial on efficacy of oligomeric formula (HINE E-GEL(R)) versus polymeric formula (MEIN(R)) enteral nutrition after esophagectomy for esophageal cancer with gastric tube reconstruction. Dis Esophagus 2019, 32(5).

13. van der Sluis PC, van der Horst S, May AM, Schippers C, Brosens LAA, Joore HCA, Kroese CC, Haj Mohammad N, Mook S, Vleggaar FP et al: Robot-assisted Minimally Invasive Thoracolaparoscopic Esophagectomy Versus Open Transthoracic Esophagectomy for Resectable Esophageal Cancer: A Randomized Controlled Trial. Ann Surg 2019, 269(4):621-630.

14. Kanekiyo S, Takeda S, Iida M, Nishiyama M, Kitahara M, Shindo Y, Tokumitsu Y, Tomochika S, Tsunedomi R, Suzuki N et al: Efficacy of perioperative immunonutrition in esophageal cancer patients undergoing esophagectomy. Nutrition 2019, 59:96-102.

15. Valkenet K, Trappenburg JCA, Ruurda JP, Guinan EM, Reynolds JV, Nafteux P, Fontaine M, Rodrigo HE, van der Peet DL, Hania SW et al: Multicentre randomized clinical trial of inspiratory muscle training versus usual care before surgery for oesophageal cancer. Br J Surg 2018, 105(5):502-511.

16. Li B, Hu H, Zhang Y, Zhang J, Miao L, Ma L, Luo X, Zhang Y, Ye T, Li H et al: Three-field versus two-field lymphadenectomy in transthoracic oesophagectomy for oesophageal squamous cell carcinoma: short-term outcomes of a randomized clinical trial. Br J Surg 2020, 107(6):647-654.

17. Berkelmans GHK, Fransen LFC, Dolmans-Zwartjes ACP, Kouwenhoven EA, van Det MJ, Nilsson M, Nieuwenhuijzen GAP, Luyer MDP: Direct Oral Feeding Following Minimally Invasive Esophagectomy (NUTRIENT II trial): An International, Multicenter, Open-label Randomized Controlled Trial. Ann Surg 2020, 271(1):41-47.

18. Mariette C, Markar SR, Dabakuyo-Yonli TS, Meunier B, Pezet D, Collet D, D'Journo XB, Brigand C, Perniceni T, Carrere N et al: Hybrid Minimally Invasive Esophagectomy for Esophageal Cancer. N Engl J Med 2019, 380(2):152-162.

19. Tao Z, Zhang Y, Zhu S, Ni Z, You Q, Sun X, Xu D: A Prospective Randomized Trial Comparing Jejunostomy and Nasogastric Feeding in Minimally Invasive McKeown Esophagectomy. J Gastrointest Surg 2020, 24(10):2187-2196.

20. Sasaki K, Omoto I, Uchikado Y, Okumura H, Noda M, Tsuruda Y, Kita Y, Arigami T, Mori S, Kurahara H et al: Comparison of greater curvature and lesser curvature circular-stapled esophagogastrostomy after esophagectomy in patients with esophageal cancer: a prospective randomized controlled trial. Surg Today 2021, 51(4):575-581.

21. Sugimura K, Miyata H, Tanaka K, Makino T, Takeno A, Shiraishi O, Motoori M, Yamasaki M, Kimura Y, Hirao M et al: Multicenter Randomized Phase 2 Trial Comparing Chemoradiotherapy and Docetaxel Plus 5-Fluorouracil and Cisplatin Chemotherapy as Initial Induction Therapy for Subsequent Conversion Surgery in Patients With Clinical T4b Esophageal Cancer: Short-term Results. Ann Surg 2021, 274(6):e465-e472.

22. Zhong J, Zhang S, Li C, Hu Y, Wei W, Liu L, Wang M, Hong Z, Long H, Rong T et al: Active cycle of breathing technique may reduce pulmonary complications after esophagectomy: A randomized clinical trial. Thorac Cancer 2022, 13(1):76-83.

23. Shi K, Qian R, Zhang X, Jin Z, Lin T, Lang B, Wang G, Cui D, Zhang B, Hua X: Video-assisted mediastinoscopic and laparoscopic transhiatal esophagectomy for esophageal cancer. Surg Endosc 2022, 36(6):4207-4214.

24. Yang Y, Li B, Yi J, Hua R, Chen H, Tan L, Li H, He Y, Guo X, Sun Y et al: Robot-assisted Versus Conventional Minimally Invasive Esophagectomy for Resectable Esophageal Squamous Cell Carcinoma: Early Results of a Multicenter Randomized Controlled Trial: the RAMIE Trial. Ann Surg 2022, 275(4):646-653.

25. van Workum F, Verstegen MHP, Klarenbeek BR, Bouwense SAW, van Berge Henegouwen MI, Daams F, Gisbertz SS, Hannink G, Haveman JW, Heisterkamp J et al: Intrathoracic vs Cervical Anastomosis After Totally or Hybrid Minimally Invasive Esophagectomy for Esophageal Cancer: A Randomized Clinical Trial. JAMA Surg 2021, 156(7):601-610.

26. Wang H, Tang H, Fang Y, Tan L, Yin J, Shen Y, Zeng Z, Zhu J, Hou Y, Du M et al: Morbidity and Mortality of Patients Who Underwent Minimally Invasive Esophagectomy After Neoadjuvant Chemoradiotherapy vs Neoadjuvant Chemotherapy for Locally Advanced Esophageal Squamous Cell Carcinoma: A Randomized Clinical Trial. JAMA Surg 2021, 156(5):444-451.

27. Liu B, Wang W, Liang T: Clinical observation of modified gastric tube in middle and lower thoracic esophageal carcinoma surgery. J Cardiothorac Surg 2019, 14(1):146.

28. Kulkarni A, Mulchandani JG, Sadat MS, Shetty N, Shetty S, Kumar MP, Kudari A: Robot-assisted versus video-assisted thoraco-laparoscopic McKeown's esophagectomy for esophageal cancer: a propensity score-matched analysis of minimally invasive approaches. J Robot Surg 2022, 16(6):1289-1297.

29. Fabbi M, van Berge Henegouwen MI, Fumagalli Romario U, Gandini S, Feenstra M, De Pascale S, Gisbertz SS: End-to-side circular stapled versus side-to-side linear stapled intrathoracic esophagogastric anastomosis following minimally invasive Ivor-Lewis esophagectomy: comparison of short-term outcomes. Langenbecks Arch Surg 2022, 407(7):2681-2692.

30. Hayes N, Shaw IH, Raimes SA, Griffin SM: Comparison of conventional Lewis-Tanner two-stage oesophagectomy with the synchronous two-team approach. Br J Surg 1995, 82(1):95-97.

31. Bruns C, Schäfer H, Wolfearten B, Pichlmaier H: Influence of surgical trauma on natural killer cell activity in esophageal carcinoma following transmediastinal dissection compared with transthoracic en bloc resection. Langenbecks Arch Chir 1996, 381(3):175-181.

32. Fransen LFC, Janssen T, Aarnoudse M, Nieuwenhuijzen GAP, Luyer MDP: Direct Oral Feeding After a Minimally Invasive Esophagectomy: A Single-Center Prospective Cohort Study. Ann Surg 2022, 275(5):919-923.
